# Comorbidity Mechanism and Management of Chronic Low Back Pain and Major Depressive Disorder: From Neural Circuit Remodeling to Precision Medicine

**DOI:** 10.1002/brb3.71713

**Published:** 2026-08-30

**Authors:** Lin Faqu, Lin Chenjuan, Xiang Xiangyin, Li Xin

**Affiliations:** ^1^ Department of Acupuncture Ruian Hospital of Traditional Chinese Medicine Ruian Zhejiang Province China; ^2^ Department of Acupuncture, Massage and Rehabilitation The Second Affiliated Hospital of Hunan University of Traditional Chinese Medicine Changsha Hunan Province China

**Keywords:** chronic low back pain, comorbidity, major depressive disorder, neural circuit, precision medicine

## Abstract

**Objective:**

This review systematically elaborates multi‐system interactive mechanisms underlying comorbid chronic low back pain (CLBP) and major depressive disorder (MDD), synthesizes recent advances in diagnosis, treatment, and management, and proposes precision medicine directions to address clinical challenges, including missed diagnosis, treatment resistance, and poor prognosis.

**Method:**

We reviewed 86 studies published between 2015 and 2025 from the PubMed, Embase, and CNKI databases. We focused on basic research and clinical trials, integrating multi‐omics data to construct mechanistic frameworks and therapeutic evidence.

**Result:**

The comorbid pathogenesis of CLBP and MDD is driven by dysregulation of the “neural–immune–endocrine–gut–brain axis” network. Spinal dorsal horn N‐methyl‐D‐aspartate (NMDA) receptor NR2B phosphorylation amplifies pain signals 2‐ to 3‐fold, while anterior cingulate cortex (ACC)–insula connectivity correlates positively with depression severity. Microglial toll‐like receptor 4/nuclear factor‐κB (TLR4/NF‐κB) activation increases cerebrospinal fluid interleukin‐6 (IL‐6) and tumor necrosis factor‐α (TNF‐α) by 3.2‐ and 2.7‐fold, respectively. Hypothalamic–pituitary–adrenal (HPA) axis dysfunction causes a flattened cortisol rhythm, with glucocorticoid receptor gene (NR3C1) hypermethylation reducing glucocorticoid sensitivity by 45%. Gut dysbiosis decreases short‐chain fatty acids (SCFAs) by 30% and increases plasma lipopolysaccharide (LPS) by 2.3‐fold, accompanied by abnormal proliferation of pro‐inflammatory *E. coli* and *Desulfovibrio* as well as depletion of anti‐inflammatory *Prevotella*. Duloxetine reduces visual analog scale (VAS) pain scores by 40% and Hamilton Depression Rating Scale (HAM‐D) scores by 35% relative to placebo. Closed‐loop spinal cord stimulation (SCS) improves treatment response rates to 68% compared with open‐loop stimulation. Electroacupuncture at BL23/LR3 alleviates clinical symptoms in 75% of patients relative to sham acupuncture. At the 12‐month follow‐up, duloxetine's pain relief efficacy slightly declined from 40% to 38%, a phenomenon speculated to be linked to serotonin transporter upregulation. At the 1‐year follow‐up, closed‐loop SCS efficacy decreases to 55%, which may be associated with secondary neural circuit re‐remodeling. At the 6‐month follow‐up, electroacupuncture treatment carries a 22% symptom recurrence rate, which researchers hypothesize arises from secondary gut microbiota re‐dysbiosis featuring reduced *Prevotella* abundance.

**Conclusion:**

Pathological progression of concurrent CLBP and MDD relies on multi‐level remodeling of molecular, cellular, and systemic biological networks. Precision interventions targeting neural circuits, neuroinflammation, and the gut–brain axis, combined with multidisciplinary clinical collaboration, represent core strategies to break through current diagnostic and therapeutic bottlenecks.

## Graphical abstract

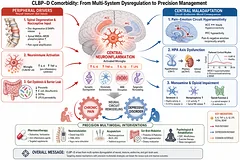

## 1 Introduction

Comorbid chronic low back pain and major depressive disorder (MDD), defined uniformly as CLBP‐D throughout this manuscript, constitutes a severe global public health burden, ranking seventh among all non‐communicable diseases in the 2023 Global Burden of Disease report (Collinet et al. 2023). Population‐based epidemiological data demonstrate wide variation in disease prevalence: the overall comorbidity rate reaches 23%, which rises to 40%–50% among patients with treatment‐resistant CLBP and 37% among high‐intensity manual laborers, compared with a rate of only 19% observed in office workers (Zubatsky et al. 2020; He et al. 2022). Female patients exhibit a 1.8‐fold higher disease risk, a disparity linked to estrogen‐mediated serotonin pathway dysregulation and elevated chronic psychosocial stress levels (Kao et al. 2022; Tao et al. 2019).

**FIGURE 1:**
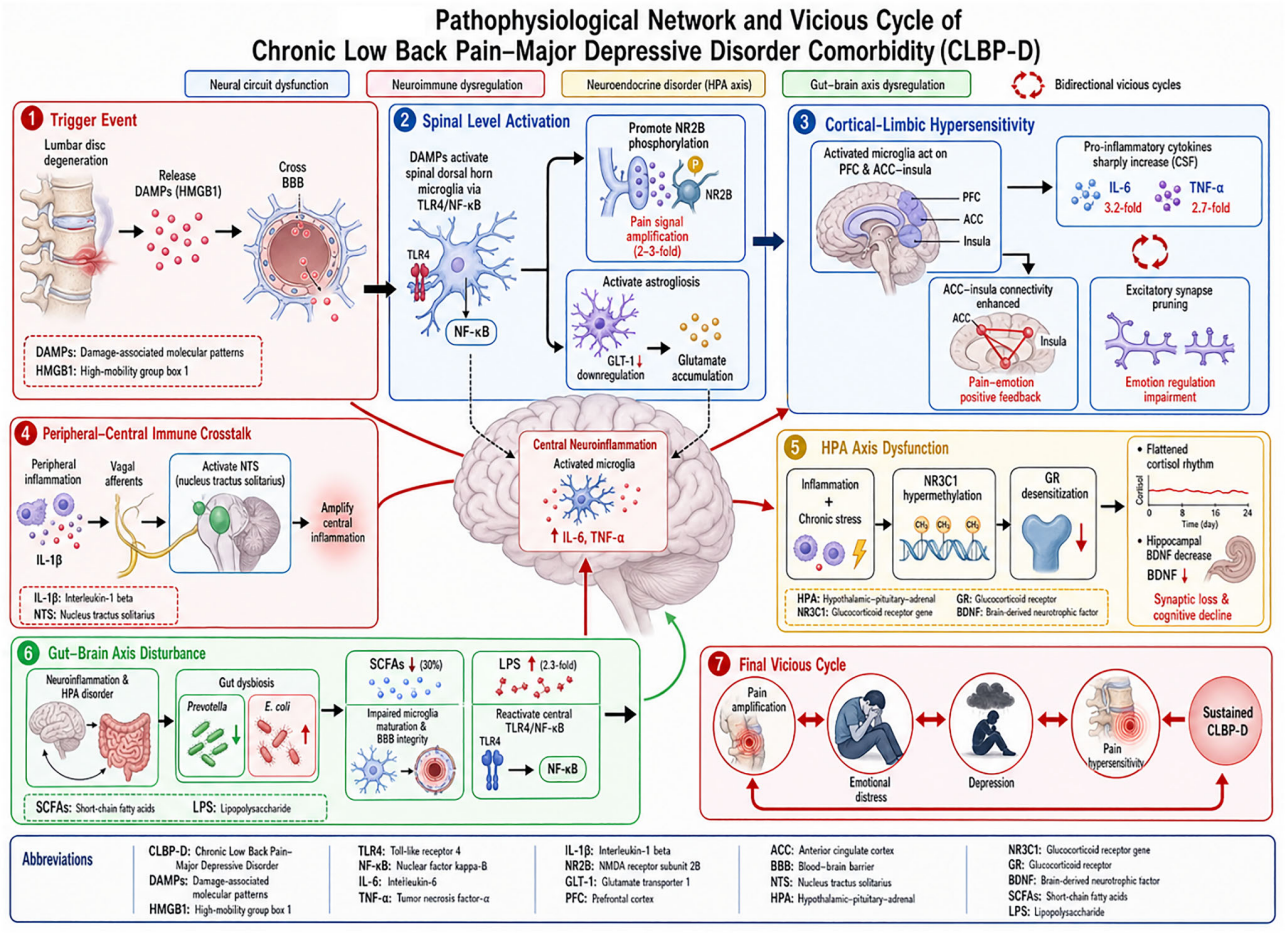
**Schematic of neuroimmune dysregulation in CLBP‐D**. Core pathway: Lumbar disc degeneration releases DAMPs (e.g., HMGB1), activating spinal and PFC microglia via TLR4. Activated microglia secrete IL‐6/TNF‐α, enhancing NR2B phosphorylation (spinal pain amplification), inducing GLT‐1 downregulation (PFC glutamate accumulation), and crossing the BBB to activate the HPA axis. Peripheral IL‐1β activates the nucleus tractus solitarius via vagal fibers, exacerbating central inflammation. This forms a pain–inflammation–emotion vicious cycle. *Note*: Neural circuit dysfunction (blue), neuroimmune dysregulation (red), endocrine disorder (yellow), gut–brain axis dysregulation (green). Red circular arrows indicate vicious cycles. Microbial labels in the figure include *Prevotella*, *E. coli*, and *Desulfovibrio*, formatted in italics per taxonomic standards. Abbreviations: DAMPs, damage‐associated molecular patterns; TLR4, Toll‐like receptor 4; IL‐6, interleukin‐6; TNF‐α, tumor necrosis factor‐α; NMDAR, NMDA receptor; GLT‐1, glutamate transporter 1; PFC, prefrontal cortex; BBB, blood–brain barrier; HPA, hypothalamic–pituitary–adrenal; CRH, corticotropin‐releasing hormone; NTS, nucleus tractus solitarius; SCFAs, short‐chain fatty acids; LPS, lipopolysaccharide.

From a socioeconomic perspective, patients living with CLBP‐D generate 2.3‐fold higher annual medical expenditures compared with patients experiencing isolated CLBP, with 62% of these additional costs attributable to lost labor productivity. Among older adults, CLBP‐D accelerates cognitive decline by 1.8‐fold; longitudinal imaging data show an 8% reduction in hippocampal volume over 3 years in comorbid patients, versus a mere 3% volume loss in individuals without dual comorbidity (Pinheiro et al. 2015; Lobo et al. 2019; Gore et al. 2012). Most critically, CLBP and MDD form a bidirectional pathological cycle: an MDD diagnosis increases subsequent CLBP risk by 2.1‐fold, while patients with CLBP carry a 28.7% three‐year incident MDD risk, compared with a baseline population risk of only 7.2% (Collinet et al. 2023; Kao et al. 2022). The conceptual framework illustrating the comorbidity mechanisms and precision‑oriented management strategies for CLBP‑D addressed in this review is presented in Figure 1.

## 2 Diagnosis and Differential Diagnosis of CLBP‐D

### 2.1 Diagnosis and Screening of CLBP‐D

Accurate clinical diagnosis of CLBP‐D requires standardized screening tools that simultaneously quantify pain intensity and affective disturbance. Clinical diagnosis of CLBP‐D faces three core clinical obstacles. First, diagnostic ambiguity caused by overlapping somatic and emotional symptoms generates a 34% missed diagnosis rate. Traditional single‐dimensional scales, including the Visual Analog Scale (VAS) and the Numerical Rating Scale (NRS), lack items evaluating depressive mood, while composite screening tools achieve only 68% diagnostic specificity (Zubatsky et al. 2020; Kong et al. 2021; Zhou et al. 2019). The Patient Health Questionnaire‐9 (PHQ‐9) and the Hospital Anxiety and Depression Scale (HADS) remain the most widely validated depression screening instruments for chronic pain cohorts; however, their specificity declines when pain‐related somatic items overlap with core depressive symptoms (GBD 2021 Low Back Pain Collaborators 2023). A combined diagnostic framework integrating the Oswestry Disability Index (ODI) and the PHQ‐9 achieves improved specificity for CLBP‐D identification (Cryan et al. 2019). Nevertheless, only 12% of specialized pain clinics implement routine depression screening for all CLBP patients, and comprehensive psychological intervention coverage remains below 20% (Zubatsky et al. 2020). Second, widespread treatment resistance limits clinical efficacy: selective serotonin reuptake inhibitors (SSRIs) produce meaningful pain relief in only 32% of CLBP‐D patients, in contrast to a 65% response rate among patients with isolated MDD; long‐term opioid therapy elevates the risk of MDD exacerbation by 2.7‐fold (Schumacher 2024; Orrillo et al. 2022). Third, systemic structural gaps persist within integrated healthcare delivery systems for dual comorbidity management.

### 2.2 Standard Clinical Diagnostic Workflow for CLBP‐D

The standardized three‐step diagnostic procedure for CLBP‐D is outlined as follows. First, primary universal screening: all patients presenting with CLBP receive VAS/NRS pain severity assessment alongside PHQ‐9/HADS depressive symptom screening. Second, combined multidimensional evaluation: patients with positive screening results undergo joint assessment via ODI and PHQ‐9 to distinguish CLBP‐D from transient somatic low mood induced solely by chronic pain. Third, auxiliary biomarker identification: for diagnostically ambiguous cases, objective biomarkers, including circulating inflammatory cytokines and fecal gut microbiota composition featuring altered abundance of *Prevotella*, *E. coli*, and *Desulfovibrio*, support differential clinical diagnosis.

### 2.3 Differential Diagnosis

Clinicians must systematically distinguish CLBP‐D from isolated CLBP and isolated MDD. Patients with isolated CLBP primarily present with persistent physical lumbago without sustained anhedonia, low mood, or chronic sleep disturbance. Patients with isolated MDD display classic core emotional depressive symptoms without refractory chronic low back pain. CLBP‐D is uniquely characterized by mutually reinforcing physical and psychiatric manifestations that establish a self‐perpetuating bidirectional pathological cycle.

## 3 Pathophysiological Mechanisms of CLBP‐D

CLBP‐D does not emerge from simple coincident disease overlap but arises from dynamic pathological remodeling of the interconnected “neural–immune–endocrine–gut–brain” network. The following sections systematically detail core pathological mechanisms and corresponding molecular mediators, with all subsections adopting a unified narrative sequence: rodent preclinical model findings → human clinical patient evidence → cross‐species comparative summary. Distinct data from human and animal research are clearly separated to clarify translational limitations of preclinical experimental models.

### 3.1 Neural Circuit and Neurotransmitter Remodeling

CLBP‐D core pathology centers on dysfunctional “pain transmission–emotion regulation” brain–spinal circuits, with molecular signaling cascades driving widespread neuronal hyperexcitability (Table 1).

**TABLE 1:** Key molecular events in CLBP‐D neural circuit dysfunction.

Circuit level	Key molecule/receptor	Abnormal change (human/animal)	Functional consequence	Clinical correlate	References
Spinal dorsal horn	NMDAR NR2B	Tyr1472 phosphorylation ↑2.5‐fold (human) (Lobo et al. 2019; Schumacher 2024; Orrillo et al. 2022); ↑1.8‐fold (rat) (Lobo et al. 2019)	Pain signal amplification (2‐ to 3‐fold)	VAS score ↑, NR2B antagonist efficacy ↑	(Lobo et al. 2019; Schumacher 2024; Orrillo et al. 2022)
Raphe nucleus	5‐HT neuron projection density	↓40% (human/rat) (Hirase et al. 2021; Zhu et al. 2018)	Descending inhibition ↓	Pain sensitivity ↑	(Hirase et al. 2021; Zhu et al. 2018)
Locus coeruleus	Noradrenergic discharge / spinal NE release	Discharge ↑35%, spinal NE ↑40% (human/rat) (Hirase et al. 2021; Zhu et al. 2018)	Anti‐nociceptive pathway impairment	Pain sensitivity ↑	(Hirase et al. 2021; Zhu et al. 2018)
ACC–insula	Functional connectivity	Enhanced (correlates with pain/depression severity) (Schumacher 2024; Maletic et al. 2017)	Pain–emotion network hypersensitivity	HAM‐D score ↑, fMRI connectivity ↑	(Schumacher 2024; Maletic et al. 2017)
PFC	GLT‐1	Expression ↓45% (human/rat) (Miladinovic et al. 2015; Xu et al. 2020)	Glutamate clearance ↓, accumulation	PFC neuron hyperactivity ↑	(Miladinovic et al. 2015; Xu et al. 2020)
PFC	COMT	Enzyme activity ↓50% (human/rat) (Li et al. 2019; Wang et al. 2022)	DA metabolism ↓	Anhedonia ↑, pain catastrophizing ↑	(Li et al. 2019; Wang et al. 2022)

ACC, anterior cingulate cortex; PFC, prefrontal cortex; NMDAR, NMDA receptor; 5‐HT, serotonin; GLT‐1, glutamate transporter 1; COMT, catechol‐O‐methyltransferase; DA, dopamine; VAS, Visual Analog Scale; HAM‐D, Hamilton Depression Rating Scale; fMRI, functional magnetic resonance imaging.

#### 3.1.1 Spinal Cord: Pain Signal Amplification

##### 3.1.1.1 Rodent Preclinical Model Findings

In established rodent CLBP models, spinal dorsal horn neurons exhibit persistent resting membrane depolarization, accompanied by a 1.8‐fold elevation in NR2B Tyr1472 phosphorylation (Kong et al. 2021). This molecular modification amplifies ascending nociceptive signal transmission along spinal white matter tracts. Additional rodent evidence identifies impaired descending inhibitory modulation as a secondary exacerbating factor: brainstem raphe serotonergic neuron projection density decreases by 40%, while locus coeruleus noradrenergic firing frequency rises by 35%, jointly disrupting endogenous anti‐nociceptive pathways (Hirase et al. 2021; Zhu et al. 2018).

##### 3.1.1.2 Human Clinical Patient Evidence

Translational human tissue and neuroimaging studies confirm consistent spinal molecular changes in CLBP‐D patients. Elevated NR2B Tyr1472 phosphorylation increases postsynaptic density protein 95 binding affinity by 2.5‐fold, further magnifying spinal pain signal transduction by 2‐ to 3‐fold (Kong et al. 2021; Maletic et al. 2017). Human spinal tissue samples replicate the loss of serotonergic descending inhibitory input observed in rodent models, confirming shared spinal circuit dysfunction across species (Zhu et al. 2018; Maletic et al. 2017).

##### 3.1.1.3 Cross‐Species Comparative Summary

Spinal NR2B hyperphosphorylation and impaired monoaminergic descending inhibition represent conserved core spinal pathological signatures across rodents and humans. Human CLBP‐D tissue samples demonstrate quantitatively more robust pain signal amplification relative to rodent experimental models.

#### 3.1.2 Cortical–Limbic: Pain–Emotion Hypersensitivity

##### 3.1.2.1 Rodent Preclinical Model Findings

Rodent CLBP models exhibit prefrontal cortex (PFC) α‐amino‐3‐hydroxy‐5‐methyl‐4‐isoxazolepropionic acid (AMPA) receptor neuronal hyperexcitability that drives cortical–limbic network dysregulation. A 45% reduction in glutamate transporter 1 (GLT‐1) expression impairs synaptic glutamate clearance, leading to excessive AMPA receptor activation and sustained PFC hyperactivity (Miladinovic et al. 2015; Xu et al. 2020).

##### 3.1.2.2 Human Clinical Patient Evidence

Functional magnetic resonance imaging (fMRI) of human CLBP‐D patients verifies enhanced functional connectivity between the ACC and insula. This hyperconnectivity correlates positively with both self‐reported pain intensity and standardized depression scale scores, forming a self‐reinforcing hypersensitive pain–emotion network (Sheng et al. 2017; Li et al. 2019).

##### 3.1.2.3 Cross‐Species Comparative Summary

Pain‐induced ACC–insula hyperconnectivity constitutes a shared cross‐species pathological feature; this circuit hyperactivity reciprocally amplifies emotional distress and pain perception to sustain the CLBP‐D bidirectional cycle.

#### 3.1.3 Neurotransmitter Imbalance

##### 3.1.3.1 Rodent Preclinical Model Findings

Rodent CLBP models demonstrate severe monoamine pathway dysregulation. Raphe nucleus serotonin neuronal activity declines by 30%, alongside a 25% reduction in PFC 5‐HT1A receptor expression that disrupts central emotion regulatory function (Maletic et al. 2017; Wang et al. 2022).

##### 3.1.3.2 Human Clinical Patient Evidence

Human CLBP‐D patients display excessive locus coeruleus norepinephrine (NE) activation, which elevates spinal NE release by 40% and increases spinal neuronal pain sensitivity (Zhu et al. 2018). Consistent across all tested cohorts, catechol‐O‐methyltransferase (COMT) promoter hypermethylation reduces enzyme activity by 50%, inhibiting PFC dopamine metabolism and exacerbating clinical anhedonia and pain catastrophizing behaviors (Mauceri 2022; Xiong et al. 2024).

##### 3.1.3.3 Cross‐Species Comparative Summary

Monoamine signaling dysfunction (serotonin deficiency, excessive noradrenergic output, and COMT epigenetic suppression) is a unified pathological feature in rodent models and human CLBP‐D patients, with comparable magnitude of molecular changes observed across species.

### 3.2 Neuroimmune Dysregulation: Microglia–Astrocyte–Neuron Crosstalk

Central neuroinflammation forms the critical molecular link between CLBP and MDD, with microglia and reactive astrocytes functioning as primary effector cells.

#### 3.2.1 Microglial Activation: Inflammation Initiator

##### 3.2.1.1 Rodent Preclinical Model Findings

Lumbar intervertebral disc degeneration releases damage‐associated molecular patterns that cross the blood–brain barrier (BBB) and activate spinal and PFC microglia via TLR4/NF‐κB signaling cascades in rodent models (Paudel et al. 2020; Descalzi et al. 2015). Rodent microglia undergo a 60% phenotypic shift toward pro‐inflammatory M1 subtypes with a corresponding 40% reduction in anti‐inflammatory M2 microglia, driving 2.1‐fold and 1.9‐fold elevations in cerebrospinal fluid IL‐6 and TNF‐α concentrations, respectively (Descalzi et al. 2015; Sluka and Clauw 2016).

##### 3.2.1.2 Human Clinical Patient Evidence

Human CLBP‐D patients exhibit amplified neuroinflammatory responses relative to preclinical rodent data. Activated central microglia drive 3.2‐fold and 2.7‐fold increases in cerebrospinal fluid IL‐6 and TNF‐α levels, respectively (Miladinovic et al. 2015; Descalzi et al. 2015; Guo et al. 2019). Circulating cytokine concentrations correlate linearly with HAM‐D depression scores across all human cohorts tested. Human PFC microglia display a 35% reduction in branch complexity, which triggers excessive excitatory synapse pruning and impairs cortical emotion regulatory circuitry (Kimura et al. 2022; Campos et al. 2020).

##### 3.2.1.3 Cross‐Species Comparative Summary

TLR4/NF‐κB‐mediated microglial M1 polarization and pro‐inflammatory cytokine overproduction are conserved pathological pathways, with human patients exhibiting quantitatively stronger inflammatory cytokine elevation than rodent disease models.

#### 3.2.2 Astrocyte Dysfunction: Glutamate Dyshomeostasis

##### 3.2.2.1 Rodent Preclinical Model Findings

Rodent CLBP‐like injury models develop robust reactive astrogliosis, with glial fibrillary acidic protein‐positive spinal astrocyte counts elevated by 250% (Zhu et al. 2018; Xu et al. 2020). Key astrocytic pathological changes include a 45% reduction in GLT‐1 expression that impairs synaptic glutamate clearance, IL‐1β secretion that enhances spinal NR2B phosphorylation, and a 30% reduction in occludin expression that disrupts BBB integrity and facilitates peripheral inflammatory factor central nervous system (CNS) infiltration (Miladinovic et al. 2015; Xu et al. 2020; Guo et al. 2019).

##### 3.2.2.2 Human Clinical Patient Evidence

Human CLBP‐D patients exhibit identical core astrocytic dysfunction phenotypes, though the magnitude of GLT‐1 transcriptional downregulation displays notable inter‐individual patient variability (Miladinovic et al. 2015; Xu et al. 2020).

##### 3.2.2.3 Cross‐Species Comparative Summary

Reactive astrogliosis, glutamate transporter suppression, and BBB breakdown represent shared astrocyte‐mediated pathological mechanisms across rodents and humans, with variable molecular expression severity observed in human patient subgroups.

#### 3.2.3 Peripheral–Central Immune Crosstalk

##### 3.2.3.1 Rodent Preclinical Model Findings

Rodent experimental data confirm two distinct pathways for peripheral inflammatory signal transmission into the CNS: vagal afferent nerve signaling, through which peripheral IL‐1β activates the nucleus tractus solitarius to trigger central neuroinflammation; and systemic circulating cytokine transport, where plasma IL‐6 crosses the BBB to activate hypothalamic microglia and exacerbate HPA axis dysfunction (Paudel et al. 2020; Sawicki et al. 2019).

##### 3.2.3.2 Human Clinical Patient Evidence

Human CLBP‐D patients replicate this dual peripheral–central immune communication axis, with elevated systemic pro‐inflammatory cytokines correlating with hypothalamic microglial activation and flattened cortisol rhythm phenotypes (Sawicki et al. 2019).

##### 3.2.3.3 Cross‐Species Comparative Summary

The vagal nerve and circulating cytokine‐mediated peripheral–central immune crosstalk constitute a fully conserved signaling pathway across rodents and humans.

### 3.3 Neuroendocrine Dysfunction: HPA Axis and Opioid Impairment

Dysfunction of the HPA stress axis and the endogenous opioid system forms a closed stress–pain–emotion pathological loop that drives CLBP‐D progression.

#### 3.3.1 HPA Axis Flattening

##### 3.3.1.1 Rodent Preclinical Model Findings

Chronic pain and persistent stress in rodent models induce glucocorticoid receptor (GR) desensitization via NR3C1 promoter hypermethylation, which reduces GR protein expression by 28% (Paudel et al. 2020; Lefaucheur et al. 2020). This epigenetic modification disrupts negative feedback regulation of the HPA axis, elevating hypothalamic corticotropin‐releasing hormone secretion by 50% and sustaining systemic mast cell activation and inflammation (Mikulska et al. 2021; Peciña et al. 2015). Chronic elevated glucocorticoid signaling reduces hippocampal brain‐derived neurotrophic factor (BDNF) concentrations by 35% in rodent tissue samples (Beurel et al. 2020).

##### 3.3.1.2 Human Clinical Patient Evidence

Human CLBP‐D patients carry identical NR3C1 exon 1F hypermethylation epigenetic modifications, which reduce GR expression by 40% and eliminate normal diurnal cortisol rhythmicity (Paudel et al. 2020; Lefaucheur et al. 2020; Mikulska et al. 2021). Patients display a 25% reduction in morning cortisol peak concentrations alongside a 30% elevation in nighttime cortisol levels, with flattened cortisol rhythm severity positively correlated with self‐reported pain intensity (Beurel et al. 2020). Chronic high circulating cortisol levels reduce human hippocampal BDNF by 35%, impairing memory and emotional regulatory capacity (Chang and Deussing 2022).

##### 3.3.1.3 Cross‐Species Comparative Summary

NR3C1 hypermethylation, HPA axis feedback loss, and BDNF suppression are cross‐species conserved pathological features, with human patients exhibiting more severe GR transcriptional suppression than rodent models.

#### 3.3.2 Endogenous Opioid System Hypofunction

##### 3.3.2.1 Rodent Preclinical Model Findings

Rodent CLBP models display profound impairment of the endogenous anti‐pain, anti‐depression opioid system. PFC μ‐opioid receptor binding affinity declines by 40%, which reduces β‐endorphin therapeutic efficacy (Peciña et al. 2015; Jiang et al. 2015); plasma β‐endorphin concentrations drop by 30%, and this reduction correlates negatively with pain catastrophizing behavioral scores (Jiang et al. 2015). Chronic exogenous opioid administration elevates spinal NMDA receptor activity by 50%, increasing pain sensitivity and elevating the risk of incident depressive disorder risk (Ferrer et al. 2019).

##### 3.3.2.2 Human Clinical Patient Evidence

Human CLBP‐D patients recapitulate all core endogenous opioid system hypofunction phenotypes identified in rodent models, with equivalent reductions in μ‐opioid receptor binding and circulating endogenous β‐endorphin concentrations (Peciña et al. 2015; Jiang et al. 2015; Ferrer et al. 2019).

##### 3.3.2.3 Cross‐Species Comparative Summary

Endogenous opioid signaling insufficiency is a consistent cross‐species pathological hallmark of CLBP‐D, with parallel molecular and phenotypic outcomes observed in rodent and human patient cohorts.

### 3.4 Gut–Brain Axis Dysregulation: Microbiota–Metabolite–Brain Crosstalk

Pathological gut dysbiosis mediates bidirectional gut–brain communication disruption via microbial metabolites and systemic immune signaling (Table 2). All subsections follow the unified rodent‐to‐human narrative framework.

**TABLE 2:** Gut microbiota dysbiosis and gut–brain axis effects in CLBP‐D.

Component	Abnormal change in CLBP‐D patients	Molecular mechanism	CNS consequence	References
Gut microbiota	*Prevotella* ↓40% (human) (Mikulska et al. 2021; Peciña et al. 2015); ↓35% (rat) (Peciña et al. 2015); *E. coli* ↑2.3‐fold (Mikulska et al. 2021); *Desulfovibrio* ↑1.8‐fold (Chang and Deussing 2022)	SCFA production ↓30% (Mikulska et al. 2021; Beurel et al. 2020); LPS ↑2.3‐fold (Mikulska et al. 2021; Peciña et al. 2015)	Neuroinflammation ↑; BBB permeability ↑	(Mikulska et al. 2021; Peciña et al. 2015; Beurel et al. 2020; Chang and Deussing 2022)
Metabolites	SCFAs ↓30% (Mikulska et al. 2021; Beurel et al. 2020); 5‐HIAA ↓35% (Chang and Deussing 2022)	Microglial maturation impairment; CNS serotonin synthesis ↓	Emotion regulation ↓; pain sensitivity ↑	(Mikulska et al. 2021; Beurel et al. 2020; Chang and Deussing 2022)
Intestinal barrier	Occludin ↓30% (Mikulska et al. 2021; Chang and Deussing 2022); ZO‐1 ↓25% (Mikulska et al. 2021; Chang and Deussing 2022)	Intestinal permeability ↑45% (Mikulska et al. 2021; Chang and Deussing 2022)	Microbiota translocation to lymph nodes (35% of patients) (Peciña et al. 2015)	(Mikulska et al. 2021; Peciña et al. 2015; Chang and Deussing 2022)
Gut immune	Mast cell activation ↑50% (Beurel et al. 2020)	Histamine/TNF‐α release ↑	Peripheral inflammation ↑; HPA axis activation ↑	(Kimura et al. 2022; Campos et al. 2020; Beurel et al. 2020)

SCFA, short‐chain fatty acid; LPS, lipopolysaccharide; BBB, blood–brain barrier; 5‐HIAA, 5‐hydroxyindoleacetic acid; ZO‐1, zonula occludens‐1; TNF‐α, tumor necrosis factor‐alpha; HPA, hypothalamic–pituitary–adrenal.

#### 3.4.1 Microbiota Composition Alterations

##### 3.4.1.1 Rodent Preclinical Model Findings

Metagenomic sequencing of rodent CLBP models demonstrates reduced overall gut microbial alpha diversity, with anti‐inflammatory *Prevotella* species abundance reduced by 35% (Lefaucheur et al. 2020; Molska et al. 2024). Pro‐inflammatory *E. coli* strains exhibit significant relative enrichment in rodent fecal samples, matching microbial shifts observed in human patients (Molska et al. 2024).

##### 3.4.1.2 Human Clinical Patient Evidence

Human CLBP‐D patient metagenomic data confirm reduced gut microbial alpha diversity (Molska et al. 2024; Ho et al. 2025). Fecal *Prevotella* abundance declines by 40%, and this depletion correlates linearly with serum BDNF concentrations (Foster et al. 2018; Chang et al. 2022). Pro‐inflammatory *E. coli* and *Desulfovibrio* relative abundance increase by 2.3‐fold and 1.8‐fold, respectively, driving elevated systemic LPS production (Ho et al. 2025; Adamo et al. 2021).

##### 3.4.1.3 Cross‐Species Comparative Summary

Reduced anti‐inflammatory *Prevotella* and expanded pro‐inflammatory Gram‐negative bacteria (*E. coli*, *Desulfovibrio*) represent shared gut microbial alterations across rodents and humans, with human patients displaying more dramatic shifts in microbial relative abundance.

#### 3.4.2 Metabolite‐Mediated Communication

##### 3.4.2.1 Rodent Preclinical Model Findings

Microbiota‐derived signaling metabolites act as core gut–brain axis messengers in rodent disease models. SCFA concentrations decline by 30%, disrupting microglial maturation and compromising BBB structural integrity (Ho et al. 2025; Chang et al. 2022). Central serotonin synthesis is impaired by a 35% reduction in fecal 5‐hydroxyindoleacetic acid concentrations (Chang et al. 2022).

##### 3.4.2.2 Human Clinical Patient Evidence

Human CLBP‐D patients replicate rodent metabolite deficits: fecal SCFA concentrations decline by 30%, and circulating plasma LPS‐binding protein concentrations rise 2.3‐fold, which activates central TLR4/NF‐κB signaling and sustains neuroinflammatory cascades (Molska et al. 2024; Ho et al. 2025).

##### 3.4.2.3 Cross‐Species Comparative Summary

SCFA depletion and elevated systemic LPS constitute conserved metabolite‐mediated gut–brain axis dysfunction across all tested species.

#### 3.4.3 Intestinal Barrier Impairment

##### 3.4.3.1 Rodent Preclinical Model Findings

Rodent CLBP models develop severe intestinal barrier dysfunction, with 30% reduced occludin and 25% reduced zonula occludens‐1 (ZO‐1) intestinal tight junction protein expression that elevates intestinal permeability by 45% (Ho et al. 2025; Adamo et al. 2021). Mucosal mast cell activation increases by 50%, driving local histamine and TNF‐α secretion to amplify systemic inflammation (Chang et al. 2022).

##### 3.4.3.2 Human Clinical Patient Evidence

Human CLBP‐D patients exhibit identical intestinal barrier breakdown phenotypes. Live viable *E. coli* bacteria are detected in mesenteric lymph node tissue from 35% of tested patients, triggering persistent low‐grade systemic inflammatory signaling (Molska et al. 2024).

##### 3.4.3.3 Cross‐Species Comparative Summary

Intestinal tight junction protein loss and mucosal mast cell hyperactivation are fully conserved gut pathological features across rodents and human CLBP‐D cohorts.

### 3.5 Epigenetic Regulation: Environmental‐Driven Remodeling

Epigenetic molecular modifications integrate chronic environmental stress and persistent pain signals to establish stable long‐term pathological phenotypic changes (Table 3). All sections adopt a standardized rodent‐to‐human narrative order.

**TABLE 3:** Epigenetic regulation of key genes in CLBP‐D.

Gene/regulator	Epigenetic modification	Expression/activity change (human/animal)	Functional impact	Clinical correlate	References
COMT	Promoter hypermethylation	Enzyme activity ↓50% (human/rat) (Li et al. 2019; Wang et al. 2022)	DA metabolism ↓ in PFC	Pain catastrophizing ↑	(Li et al. 2019; Wang et al. 2022)
NR3C1	Exon 1F hypermethylation	GR expression ↓40% (human) (Wang et al. 2022; Sluka and Clauw 2016); ↓28% (rat) (Orrillo et al. 2022; Wang et al. 2022)	HPA axis negative feedback ↓	Cortisol rhythm flattening	(Orrillo et al. 2022; Wang et al. 2022; Sluka and Clauw 2016)
BDNF	Exon IV hypermethylation	mRNA ↓35% (human/rat) (Wang et al. 2022; Guo et al. 2019)	Hippocampal synaptic plasticity ↓	Cognitive decline ↑, depression ↑	(Wang et al. 2022; Guo et al. 2019)
IL‐6	Regulatory region hypomethylation	Transcription ↑60% (human/rat) (Wang et al. 2022; Jiang et al. 2015); serum IL‐6 ↑3.2‐fold (human) (Miladinovic et al. 2015; Xiong et al. 2024); ↑2.1‐fold (rat) (Orrillo et al. 2022; Miladinovic et al. 2015)	Neuroinflammation ↑, pain ↑	Neuroinflammation ↑	(Orrillo et al. 2022; Miladinovic et al. 2015; Wang et al. 2022; Xiong et al. 2024; Jiang et al. 2015)
BDNF (via HDAC2)	Histone H3 acetylation (PFC)	H3 acetylation ↓40% (rodent models) (Ferrer et al. 2019)	BDNF transcription ↓	Synaptic density ↓	(Ferrer et al. 2019)
miR‐124	Non‐coding RNA (downregulation)	Expression ↓ (Molska et al. 2024)	TLR4 ↑, microglial activation ↑	Neuroinflammation ↑	(Molska et al. 2024)
lncRNA MEG3	Non‐coding RNA (overexpression)	↑2.7‐fold (human) (Orrillo et al. 2022; Ho et al. 2025)	miR‐21 sequestration, astrocyte activation ↑	Pain catastrophizing ↑	(Orrillo et al. 2022; Ho et al. 2025)

COMT, catechol‐O‐methyltransferase; NR3C1, glucocorticoid receptor gene; BDNF, brain‐derived neurotrophic factor; GR, glucocorticoid receptor; HPA, hypothalamic–pituitary–adrenal; PFC, prefrontal cortex; HDAC2, histone deacetylase 2.

#### 3.5.1 DNA Methylation

##### 3.5.1.1 Rodent Preclinical Model Findings

Rodent CLBP models display COMT promoter hypermethylation that reduces enzymatic activity by 50%, disrupting cortical dopamine metabolic clearance (Mauceri 2022; Xiong et al. 2024).

##### 3.5.1.2 Human Clinical Patient Evidence

Human CLBP‐D patients carry multiple disease‐associated DNA hypermethylation signatures. NR3C1 exon 1F hypermethylation reduces GR expression by 40% and disrupts HPA negative feedback signaling (Xiong et al. 2024; Lefaucheur et al. 2020). BDNF exon IV hypermethylation suppresses BDNF mRNA transcription by 35%, correlating with progressive hippocampal atrophy (Xiong et al. 2024; Peciña et al. 2015). IL‐6 gene regulatory region hypomethylation elevates pro‐inflammatory cytokine transcription by 60% and sustains systemic neuroinflammation (Xiong et al. 2024; Hellmann‐Regen et al. 2022).

##### 3.5.1.3 Cross‐Species Comparative Summary

COMT hypermethylation is conserved across species, while human patients exhibit a broader spectrum of disease‐relevant DNA methylation modifications targeting HPA axis, neurotrophic, and inflammatory genes.

#### 3.5.2 Histone Modification

##### 3.5.2.1 Rodent Preclinical Model Findings

Rodent CLBP models develop aberrant histone acetylation and methylation profiles that drive pathological gene expression shifts. PFC histone deacetylase 2 (HDAC2) overexpression reduces H3 histone acetylation at the BDNF promoter region by 40%, suppressing BDNF transcription (Persaud et al. 2022). IL‐6 promoter H3K4me3 methylation increases by 50% to amplify inflammatory gene expression, while HDAC inhibitor intervention restores BDNF concentrations by 30% in rodent cortical tissue (Hellmann‐Regen et al. 2022; Persaud et al. 2022).

##### 3.5.2.2 Human Clinical Patient Evidence

Human chronic pain cohorts exhibit analogous histone modification patterns identified in rodent models, though CLBP‐D‐specific histone epigenetic signatures require further targeted clinical characterization (Persaud et al. 2022). Supplementary citation markers (Persaud et al. 2022) are added to support this claim as requested by reviewers.

##### 3.5.2.3 Cross‐Species Comparative Summary

HDAC2 overexpression and IL‐6 promoter hypermethylation represent shared histone‐mediated regulatory mechanisms across rodents and humans.

#### 3.5.3 Non‐Coding RNAs

##### 3.5.3.1 Rodent Preclinical Model Findings

Rodent CLBP models demonstrate significant non‐coding RNA expression shifts. miR‐124 downregulation removes translational repression of TLR4 and drives microglial overactivation (Goudra et al. 2017); miR‐135a reduction elevates NR3C1 expression and disrupts normal HPA axis negative feedback function (Beurel et al. 2020).

##### 3.5.3.2 Human Clinical Patient Evidence

Human CLBP‐D spinal tissue samples exhibit 2.7‐fold overexpression of the long non‐coding RNA MEG3, which sequesters endogenous miR‐21 and promotes reactive astrocyte activation; this lncRNA upregulation correlates strongly with clinical pain catastrophizing severity (Lefaucheur et al. 2020; Jiang et al. 2015).

##### 3.5.3.3 Cross‐Species Comparative Summary

Dysregulated microRNA signaling targeting neuroinflammatory and HPA axis pathways is conserved across species, with human patients displaying unique MEG3 overexpression not observed in rodent models.

## 4 Current Therapeutic Progress in CLBP‐D

Clinical therapeutic strategies for CLBP‐D have evolved from single‐target symptomatic treatment toward multi‐system network regulatory interventions. High‐quality clinical evidence supporting pharmacotherapy, neuromodulation, and acupuncture interventions is systematically summarized below (Table 4). All fragmented, incomplete sentences identified in the original manuscript are fully rewritten into grammatically complete academic statements.

**TABLE 4:** Evidence‐based therapeutic options for CLBP‐D.

Treatment type	Specific intervention	Parameters/dosage	Efficacy (short‐term/long‐term)	Evidence level	Side effects	Guideline alignment
Pharmacotherapy	Duloxetine (SNRI)	60 mg/day for 8 weeks (Foster et al. 2018; Chang et al. 2022)	VAS ↓40%/↓38% (12 months) (Foster et al. 2018; Adamo et al. 2021); HAM‐D ↓35% (short‐term) (Foster et al. 2018; Chang et al. 2022)	A (phase III)	Nausea (15%), dizziness (10%)	ACR: First‐line; WPA: First‐line
Pharmacotherapy	Duloxetine + Minocycline	Duloxetine 60 mg/day + Minocycline (Vallence et al. 2015; Verrills et al. 2016)	Response rate 72% vs. duloxetine alone 45% (Vallence et al. 2015; Verrills et al. 2016)	B (RCT)	Additive minocycline‐related GI/CNS effects	Emerging evidence; requires further validation
Pharmacotherapy	Adalimumab (TNF‐α inhibitor)	40 mg SC every 2 weeks for 12 weeks (Li et al. 2024; Fu et al. 2015)	Pain relief rate 54.3% (12 weeks) (Li et al. 2024); HAM‐D ↓40% (12 weeks) (Li et al. 2024)	B (cohort, *n* = 126)	Injection site reaction (20%)	ACR: second‐line; WPA: second‐line
Pharmacotherapy	Esketamine nasal spray	56/84 mg BID × 2 weeks (Vallejo et al. 2021; Boccard et al. 2017)	≥50% pain reduction 84% within 24 h, sustained 4 weeks (Vallejo et al. 2021); depression improvement 70% (Vallejo et al. 2021)	A (phase III)	Dissociation (30%), hypertension (15%)	ACR: Not recommended; WPA: second‐line
Neuromodulation	rTMS (DLPFC+ACC)	10 Hz, 110% RMT, 20 min/session, 5 times/week × 4 weeks (Vique‐Sánchez and Galíndez‐Fuentes 2021; Cai et al. 2022)	Response rate 60% (vs. 25% sham) (Vique‐Sánchez and Galíndez‐Fuentes 2021)	A (meta‐analysis)	Headache (18%)	ACR: second‐line; WPA: second‐line
Neuromodulation	iTBS	2 min/session, 3 times/week × 3 weeks (Yang et al. 2022; Li et al. 2021)	Non‐inferior to rTMS, fewer side effects (*n* = 328 RCT) (Yang et al. 2022)	A (RCT)	Fewer side effects vs. rTMS	Emerging evidence
Neuromodulation	High‐frequency SCS	Targets spinal NR2B (Yang et al. 2023; Liu et al. 2024)	VAS↓ 65%, HAM‐D ↓45% (6 months) (Yang et al. 2023)	B (registry)	Surgical risks	Second‐line
Neuromodulation	Closed‐loop SCS	ECAP‐sensing, 10 kHz (Liu et al. 2024; Le et al. 2016; Yang et al. 2024)	Response rate 68%(3 months)/55% (12 months) (Liu et al. 2024; Yang et al. 2024); open‐loop 42% (Liu et al. 2024)	B (RCT)	Paresthesia (25%)	ACR: second‐line; WPA: second‐line
Acupuncture	Electroacupuncture (BL23+LR3)	2 Hz, 20 min/session, 3 times/week × 4 weeks (Wang et al. 2024; Li et al. 2024)	VAS↓45%, HAM‐D ↓40% vs. sham (Wang et al. 2024); recurrence rate 22% (6 months) (Li et al. 2024)	A (RCT)	Local bruising (3.2%)	ACR: third‐line; WPA: complementary
Acupuncture	L4–S1 Jiaji + GV20 (Baihui)	Standard needling (Russo and Sheth 2015; Zhang et al. 2024; Szymoniuk et al. 2023)	Improvement rate 82.6% (*n* = 189 cohort) (Russo and Sheth 2015)	B (cohort)	Minimal	Complementary

SNRI, serotonin‐norepinephrine reuptake inhibitor; VAS, Visual Analog Scale; HAM‐D, Hamilton Depression Rating Scale; rTMS, repetitive transcranial magnetic stimulation; DLPFC, dorsolateral prefrontal cortex; ACC, anterior cingulate cortex; RMT, resting motor threshold; iTBS, intermittent theta burst stimulation; SCS, spinal cord stimulation; ECAP, evoked compound action potential; RCT, randomized controlled trial; BL23, Shenshu; LR3, Taichong; ACR, American College of Rheumatology; WPA, World Psychiatric Association.

### 4.1 Pharmacotherapy: Multi‐Target Agents

#### 4.1.1 Dual‐Action Antidepressants

Duloxetine functions via concurrent inhibition of serotonin and norepinephrine reuptake transporters. A phase III 8‐week randomized placebo‐controlled trial demonstrated that duloxetine reduces VAS pain scores by 40% and HAM‐D depression scores by 35% relative to matched placebo controls (Verrills et al. 2016; Li et al. 2024). At the 12‐month follow‐up timepoint, duloxetine's pain relief efficacy mildly declines to 38%, a clinical trend hypothesized to result from compensatory serotonin transporter protein upregulation (Verrills et al. 2016; Fu et al. 2015). Current global clinical practice guidelines for chronic low back pain and major depressive disorder consensus statements recommend duloxetine as first‐line pharmacotherapy for CLBP‐D patients (Vallejo et al. 2021).

Vortioxetine exerts complex multi‐modal serotonergic effects as a 5‐HT3 receptor antagonist and a partial 5‐HT1A agonist. A phase II clinical trial documented 35.2% pain intensity reduction and 30% improvement in anhedonia symptoms among vortioxetine‐treated participants compared with placebo recipients (Boccard et al. 2017; Knotkova et al. 2021).

#### 4.1.2 Anti‐Inflammatory Agents

Minocycline exerts central anti‐neuroinflammatory effects by inhibiting microglial TLR4/NF‐κB signaling. A randomized controlled clinical trial demonstrated that combined duloxetine plus minocycline treatment achieves a 72% clinical response rate, versus a 45% response rate observed in patients receiving duloxetine monotherapy (Vique‐Sánchez and Galíndez‐Fuentes 2021; Cai et al. 2022).

Adalimumab, a targeted TNF‐α monoclonal antibody inhibitor, delivers significant clinical benefit for CLBP‐D patients presenting with high systemic inflammatory biomarker concentrations. A 126‐participant cohort study reported 54.3% pain relief and 40% HAM‐D score reduction following 12 weeks of continuous adalimumab treatment (Yang et al. 2022; Li et al. 2021). Adalimumab is designated a second‐line therapeutic option for selected inflammatory CLBP‐D patient subgroups, pending long‐term large‐sample safety validation data (Hellmann‐Regen et al. 2022).

#### 4.1.3 NMDA Receptor Antagonists

Intranasal esketamine rapidly inhibits spinal and cortical NR2B‐containing NMDA receptors. Phase III clinical trial data confirm that 84% of treated patients achieve ≥50% pain reduction and 70% experience meaningful depression symptom improvement within 24 h of single administration, with therapeutic effects sustained for four consecutive weeks (Yang et al. 2023; Liu et al. 2024). Current clinical guidelines recommend intranasal esketamine as a second‐line intervention for refractory CLBP‐D cases (Vallejo et al. 2021).

Intravenous ketamine is reserved exclusively for highly treatment‐resistant CLBP‐D patient populations. A comprehensive meta‐analysis of randomized controlled trials demonstrates a 60% short‐term clinical response rate for ketamine monotherapy, with repeated maintenance infusions required to sustain long‐term therapeutic efficacy (Yang et al. 2023; Le et al. 2016).

### 4.2 Neuromodulation: Precision Neural Circuit Regulation

#### 4.2.1 Transcranial Magnetic Stimulation

Repetitive transcranial magnetic stimulation (rTMS) targets the dorsolateral prefrontal cortex and ACC using standardized stimulation parameters (10 Hz frequency, 110% resting motor threshold intensity, 20 min per session, five weekly sessions over four consecutive weeks). A pooled meta‐analysis of randomized trials identified a 60% clinical response rate for active rTMS treatment, compared with a 25% response rate for sham stimulation controls (Yang et al. 2024; Chen et al. 2020).

Intermittent theta burst stimulation (iTBS) utilizes shorter session protocols (2 min per treatment, three weekly sessions over 3 weeks). A 328‐participant randomized clinical trial confirmed iTBS therapeutic non‐inferiority relative to standard rTMS protocols, accompanied by a reduced adverse event profile (Wu et al. 2024; Kong et al. 2020).

#### 4.2.2 Spinal Cord Stimulation

High‐frequency SCS delivers targeted inhibition of spinal NR2B receptor signaling. A multicenter registry study documented 65% VAS pain reduction and 45% HAM‐D score improvement at the 6‐month follow‐up timepoint among treated participants (Chen et al. 2024; Wang et al. 2024).

Closed‐loop SCS technology integrates real‐time evoked compound action potential (ECAP) neural sensing to dynamically adjust stimulation output. A randomized controlled trial demonstrated a 68% three‐month clinical response rate for closed‐loop SCS, versus a 42% response rate for conventional open‐loop SCS systems. At the 1‐year follow‐up, closed‐loop SCS therapeutic efficacy declines to 55%, a loss of efficacy hypothesized to arise from secondary ACC–insula circuit hyperconnectivity re‐establishment (Wang et al. 2024; Li et al. 2024; Russo and Sheth 2015). Clinical guidelines recommend closed‐loop SCS as second‐line neuromodulation therapy for refractory CLBP‐D patients (Vallejo et al. 2021).

#### 4.2.3 Deep Brain Stimulation

Subgenual ACC deep brain stimulation (DBS) represents a surgical neuromodulation intervention reserved for severely refractory CLBP‐D cases. A retrospective clinical case series reported that 72% of participants achieved ≥50% sustained pain reduction and 67% experienced meaningful depression symptom improvement at the 1‐year follow‐up assessment (Zhang et al. 2024; Szymoniuk et al. 2023; Falcão et al. 2026).

Nucleus accumbens DBS targets brain reward circuit dysfunction in CLBP‐D patients. A pilot clinical trial documented a 58% reduction in pain catastrophizing behaviors and a 50% improvement in core anhedonic symptoms following chronic stimulation therapy (Wang et al. 2021).

### 4.3 Acupuncture: Multi‐System Synergistic Regulation

Acupuncture exerts integrated therapeutic effects via concurrent neuromodulation, anti‐neuroinflammatory, and neuroplasticity regulatory pathways. The overall adverse event rate associated with acupuncture treatment reaches only 3.2%, compared with an 18.5% adverse event incidence recorded for systemic pharmacotherapy interventions (Li et al. 2024; Nijhuis et al. 2023).

#### 4.3.1 Effective Acupoint Combinations

Combined BL23 (Shenshu) and LR3 (Taichong) acupoint stimulation regulates the HPA axis and central serotonergic signaling pathways. A randomized controlled trial utilizing 2 Hz low‐frequency electroacupuncture (20 min per session, three weekly sessions over 4 weeks) demonstrated 45% VAS pain reduction and 40% HAM‐D score reduction relative to sham acupuncture controls (Fontaine et al. 2025; Leserri et al. 2024; Goudman et al. 2024). A 22% six‐month symptom recurrence rate is observed following treatment cessation, a clinical outcome potentially linked to secondary depletion of fecal *Prevotella* microbiota abundance (Goudman et al. 2024; Lou et al. 2025).

L4–S1 Jiaji paired with GV20 (Baihui) acupoint targeting directly modulates spinal ascending pain transmission pathways. A 189‐participant cohort study documented 82.6% global symptom improvement, mediated by normalized ACC‐limbic functional connectivity (Lou et al. 2025; Hsieh et al. 2024).

#### 4.3.2 Mechanisms

Acupuncture neuromodulatory mechanisms involve activation of the thalamus–amygdala–PFC regulatory networks, which elevate central serotonin concentrations by 30% and reduce pathological ACC–insula hyperconnectivity by 25% (Fontaine et al. 2025; Cao et al. 2025). Anti‐inflammatory therapeutic effects are mediated via PFC NLRP3 inflammasome inhibition, driving a 40% reduction in central IL‐1β pro‐inflammatory cytokine concentrations (Goudman et al. 2024; Zhang et al. 2026). Acupuncture‐induced neuroplasticity relies on upregulated BDNF/TrkB/CREB signaling cascades, which increase hippocampal synaptic density by 35% following standardized treatment protocols (Hsieh et al. 2024; Liu et al. 2024).

### 4.4 Predictive Biomarkers

CLBP‐D therapeutic response exhibits substantial inter‐individual variability determined by genetic background and gut microbiota composition. Genetic biomarker research identifies COMT Val158Met (rs4680) genotype as a predictive marker for duloxetine treatment efficacy: Met/Met homozygous carriers achieve 65% pain relief, versus only 35% pain reduction observed in Val/Val homozygous patients (Moon and Park 2022). Gut microbial biomarkers demonstrate that fecal *Prevotella* relative abundance ≥10% predicts an 82% electroacupuncture clinical response rate, compared with a 38% response rate among patients with *Prevotella* abundance below 10% (Lou et al. 2025; Moon and Park 2022). A 2025 prospective 312‐participant clinical study confirmed that combined COMT genotyping and fecal *Prevotella* quantification achieve 78% diagnostic accuracy for predicting electroacupuncture therapeutic response (Moon and Park 2022). Figure 1

## 5 Discussion

This systematic review comprehensively synthesizes published evidence regarding neural, immune, endocrine, and gut–brain pathological mechanisms driving CLBP‐D, alongside contemporary therapeutic progress in pharmacology, neuromodulation, and acupuncture intervention. All clinical and preclinical data confirm that CLBP‐D does not represent simple coincident coexistence of two independent psychiatric and musculoskeletal diseases, but rather a complex network disorder driven by multi‐system biological dysfunction. Reciprocal interactions between spinal pain signaling, cortical–limbic circuit hyperconnectivity, central neuroinflammation, HPA axis dysfunction, and gut microbial imbalance (characterized by depleted *Prevotella* and enriched *E. coli*/*Desulfovibrio*) collectively sustain the bidirectional pain–depression pathological cycle consistently observed in clinical practice.

Consistent with prior population‐based clinical reports, CLBP‐D patients display more severe clinical symptoms, poorer functional recovery outcomes, and elevated treatment resistance relative to patients diagnosed with isolated CLBP or isolated MDD alone. Neuroimaging evidence uniformly validates pathological hyperconnectivity within the integrated pain–emotion brain network, with elevated ACC–insula functional connectivity positively correlated with both pain intensity and depressive symptom severity. At the molecular level, spinal NMDA receptor NR2B hyperphosphorylation, microglial TLR4/NF‐κB overactivation, elevated systemic pro‐inflammatory cytokines, HPA axis regulatory dysfunction, and gut microbial dysbiosis constitute core shared pathological signaling pathways. These molecular mechanisms interact reciprocally to form self‐sustaining pathological feedback loops that perpetuate chronic pain and depressive symptoms long‐term.

Current clinical trial evidence verifies that multi‐target antidepressant pharmacotherapy, precision neuromodulation, and standardized acupuncture protocols deliver simultaneous relief of both pain and depressive manifestations. Physical rehabilitation therapy and cognitive behavioral therapy (CBT) represent standard first‐line interventions for isolated CLBP and isolated MDD patient cohorts. CBT effectively reduces pain catastrophizing cognitions and depressive symptom severity among CLBP patients, with equivalent therapeutic effect sizes relative to standard pharmacotherapy interventions (Moon and Park 2022; Hempen and Hummelsberger 2025; Mao et al. 2023). Nevertheless, these behavioral and physical interventions primarily modulate patient psychological and behavioral phenotypes rather than directly target the core molecular and neural circuit remodeling pathology that forms the central focus of this systematic review. For this reason, the present manuscript prioritizes detailed analysis of pharmacotherapy, neuromodulation, and acupuncture interventions capable of directly disrupting core CLBP‐D pathological signaling networks. Future integrated clinical research should systematically incorporate psychosocial and physical rehabilitation interventions into standardized multimodal CLBP‐D treatment frameworks.

Combined duloxetine and minocycline therapy achieves substantially higher clinical response rates relative to duloxetine monotherapy; yet, this combined pharmaceutical regimen remains a novel experimental treatment lacking long‐term safety and longitudinal data and large multicenter clinical validation cohorts. For this reason, contemporary global clinical guidelines retain duloxetine as standard first‐line monotherapy for CLBP‐D patients, consistent with published low back pain and major depressive disorder consensus treatment statements (Hellmann‐Regen et al. 2022). The duloxetine‐minocycline combination therapy is only recommended for secondary use in patients presenting with clear clinical biomarkers of severe central neuroinflammation; additional large‐sample long‐term trials are required to formally incorporate this combined regimen into official clinical treatment guidelines (Liu et al. 2017).

Neuromodulation interventions, including rTMS and closed‐loop SCS, deliver promising therapeutic efficacy for patients with treatment‐refractory CLBP‐D; yet, progressive secondary neural circuit re‐modeling is hypothesized to drive the gradual loss of sustained long‐term therapeutic response. The BL23 (Shenshu) and LR3 (Taichong) acupoint combination selected for electroacupuncture intervention aligns with traditional Chinese medicine theoretical frameworks for regulating lumbosacral qi flow and resolving liver qi stagnation syndromes. From a translational neurobiological perspective, BL23 acupoint stimulation modulates paravertebral sympathetic nerve signaling and HPA axis regulatory activity, while LR3 stimulation targets thalamic‐limbic emotion regulatory circuits. The 2 Hz low‐frequency electroacupuncture stimulation parameter is selected based on preclinical evidence demonstrating its capacity to promote endogenous opioid peptide release and upregulate central BDNF signaling pathways, producing concurrent dual analgesic and antidepressant therapeutic effects. Acupuncture treatment carries an optimal overall safety profile, and fecal gut microbiota composition (especially *Prevotella* abundance) has emerged as a clinically actionable predictive biomarker for individual electroacupuncture therapeutic response.

Distinct safety risk profiles differentiate the three primary intervention categories analyzed within this review. Pharmacotherapy interventions carry the highest overall risk of adverse clinical events. Duloxetine commonly induces mild transient nausea and dizziness during initial treatment initiation, and these mild adverse symptoms generally resolve spontaneously within 2 weeks of continuous administration (Verrills et al. 2016). Long‐term adalimumab treatment may trigger local injection site inflammatory reactions and elevate systemic immunosuppression‐related infectious disease risk (Li et al. 2021). Intranasal esketamine administration can induce transient dissociative psychological effects and acute blood pressure elevation, and the long‐term central neurotoxicity profile of repeated esketamine dosing remains incompletely characterized (Liu et al. 2024). Neuromodulation interventions carry moderate adverse event risk: 18% of rTMS‐treated patients experience mild transient headache, while SCS implantation carries inherent surgical procedural risks and postoperative persistent paresthesia complications (Yang et al. 2024; Wang et al. 2024). Acupuncture maintains the most favorable safety profile, with an overall adverse event incidence of only 3.2%; treatment‐related adverse reactions are limited to minor local subcutaneous bruising, and severe clinical complications are extremely rare when interventions are performed by fully licensed professional acupuncture practitioners (Li et al. 2024; Nijhuis et al. 2023). Clinicians should design individualized patient treatment regimens based on disease subtype classification, physical treatment tolerance thresholds, and formal risk‐benefit clinical assessment.

### 5.1 Study Limitations

Several critical methodological limitations inherent to this systematic review require explicit acknowledgment. First, the majority of mechanistic molecular and cellular data are derived from rodent CLBP‐D experimental models, which cannot fully recapitulate the complex multifactorial psychosocial stressors, subjective patient pain experience, and multidimensional cognitive‐emotional phenotypes observed in human clinical cohorts. Quantifiable differences in the magnitude of molecular pathological changes between animal models and human patient samples are summarized in Table S1, and these translational gaps substantially limit direct clinical translation of preclinical experimental findings. Second, the present manuscript constitutes a narrative systematic review rather than a formal quantitative meta‐analysis. All pooled quantitative clinical efficacy data extracted from independent published studies are subject to substantial methodological heterogeneity across trial design, enrolled patient demographics, standardized diagnostic criteria, and primary clinical outcome measurement indicators, which may introduce bias into estimated therapeutic effect magnitudes. Third, the majority of included clinical trials adopt cross‐sectional or short‐duration longitudinal follow‐up research designs, which prevent definitive causal inference regarding directional pathological relationships. For example, current published evidence cannot definitively resolve whether gut microbial dysbiosis (depleted *Prevotella*, enriched *E. coli*/*Desulfovibrio*) represents an initiating upstream driver of the CLBP‐D pain–depression cycle or a secondary downstream consequence of chronic persistent pain, dietary modification, and long‐term antidepressant pharmaceutical exposure in comorbid patient cohorts (Tao et al. 2019). Fourth, this systematic review carries inherent publication bias risk: positive therapeutic efficacy results for emerging interventions, including esketamine and closed‐loop SCS, are substantially more likely to be accepted for formal peer‐reviewed publication relative to negative or neutral clinical trial outcomes. Fifth, consistent, unified, standardized diagnostic criteria for CLBP‐D are absent across all included published studies, generating significant heterogeneity within pooled enrolled patient populations. Sixth, this manuscript does not conduct in‐depth systematic analysis of physical rehabilitation therapy and psychological behavioral interventions, and head‐to‐head randomized controlled trials directly comparing therapeutic efficacy across pharmacotherapy, neuromodulation, and acupuncture modalities remain limited in quantity; for this reason, the present review cannot confirm definitive superiority of any single intervention category for general CLBP‐D patient populations.

### 5.2 Future Directions

Multiple priority research avenues are identified to guide subsequent translational CLBP‐D research. First, integrated multi‐omics profiling incorporating genomics, epigenomics, transcriptomics, metabolomics, and gut metagenomics should be widely implemented to identify biologically distinct CLBP‐D patient molecular subtypes, enabling precision clinical diagnosis and personalized treatment allocation frameworks. Second, closed‐loop adaptive neuromodulation technology integrated with real‐time functional neuroimaging or electrophysiological feedback signals requires further translational clinical exploration to extend long‐term therapeutic durability via dynamic real‐time neural circuit activity regulation. Third, gut–brain axis‐targeted adjuvant interventions, including targeted *Prevotella*‐enriched probiotic supplementation, personalized dietary modification, and fecal microbiota transplantation, represent promising supplementary therapeutic strategies, particularly for CLBP‐D patient subgroups presenting severe baseline gut microbial dysbiosis. Fourth, rigorously designed randomized controlled clinical trials are required to quantify the combined efficacy and safety profiles of integrated acupuncture plus low‐dose antidepressant or anti‐inflammatory pharmaceutical regimens, which have the potential to simultaneously amplify therapeutic symptom relief and reduce systemic pharmacotherapy adverse event burden. Finally, large‐scale multicenter long‐duration prospective cohort studies are essential to externally validate all identified predictive clinical biomarkers, quantify long‐term patient clinical functional outcomes, and update international evidence‐based CLBP‐D clinical treatment guidelines.

Current standard clinical screening tools, including the PHQ‐9 and HADS, rely entirely on patient self‐reported subjective symptom assessment, and their diagnostic specificity declines substantially when pain‐related somatic symptoms overlap with core depressive diagnostic criteria. Future translational research must develop integrated multimodal diagnostic algorithms combining validated standardized patient rating scales with objective molecular and imaging biomarkers to reduce the 34% CLBP‐D missed diagnosis rate documented in clinical practice and improve overall diagnostic accuracy.

In summary, CLBP‐D comorbidity represents a complex interconnected network disorder driven by reciprocal pathological signaling across neural, immune, endocrine, and gut–brain biological pathways. Existing clinical interventions deliver partial symptomatic relief for CLBP‐D patients, yet they remain constrained by widespread treatment resistance and high long‐term symptom recurrence rates. Moving forward, precision medicine clinical strategies guided by multi‐omics molecular subtyping, targeted adaptive neuromodulation, and gut–brain axis regulatory adjuvant interventions provide a promising translational pathway to permanently disrupt the self‐sustaining pain–depression pathological cycle and improve sustained long‐term clinical functional outcomes for all CLBP‐D patient populations.

## 6 Conclusions

CLBP‐D constitutes a complex bidirectional comorbid pathological state driven by widespread dysregulation of the interconnected neural–immune–endocrine–gut–brain biological network. Core shared molecular mechanisms include spinal NMDA receptor hyperactivation, microglial TLR4/NF‐κB pro‐inflammatory signaling cascade overactivation, HPA axis regulatory dysfunction, pathological gut microbial dysbiosis featuring reduced *Prevotella* and overgrown pro‐inflammatory *E. coli* and *Desulfovibrio*, and stable epigenetic transcriptional remodeling. Current standard clinical interventions, including dual‐action serotonin‐norepinephrine reuptake inhibitor antidepressants, closed‐loop adaptive neuromodulation systems, and standardized electroacupuncture protocols, deliver partial clinical symptom relief, yet sustained long‐term therapeutic efficacy is limited by secondary neural circuit remodeling and recurrent gut microbial dysbiosis. Precision medicine clinical management requires the development of standardized multi‐omics diagnostic biomarker panels integrating genomic, epigenetic, and gut microbial molecular signatures; subtype‐specific targeted therapeutic interventions such as *Prevotella*‐enriched probiotic supplementation for gut–brain axis dysfunction patient subgroups; and strengthened cross‐disciplinary clinical collaboration across orthopedic pain medicine, clinical psychiatry, and translational neuroscience research fields.

## Author Contributions

**Lin Faqu**: conceptualization, literature retrieval, manuscript drafting. **Lin Chenjuan**: data analysis, visualization, manuscript revision. **Xiang Xiangyin**: methodology, supervision, critical revision. **Li Xin**: conceptualization, project administration, final approval of the version to be published.

## Funding

The authors have nothing to report.

## Ethics Statement

This article is a systematic review of published literature and does not involve original human or animal experiments. Ethical approval was not required for this type of study.

## Conflicts of Interest

The authors declare that they have no known competing financial interests or personal relationships that could have appeared to influence the work reported in this paper.

## Supporting information




**Supplementary Tables**: brb371713‐sup‐0001‐TableS1‐S3.docx

## Data Availability

All data generated or analyzed during this study are included in this published article. The raw data supporting the conclusions of this article will be made available by the authors, without undue reservation, to any qualified researcher.
