# Supplementary material for: Comorbidity Mechanism and Management of Chronic Low Back Pain and Major Depressive Disorder: From Neural Circuit Remodeling to Precision Medicine
Source: Brain Behav. 2026 Aug 30;16(9):e71713. doi: 10.1002/brb3.71713 (PMC13527223; doi:10.1002/brb3.71713)
Supplement: Supplementary file 1 [file BRB3-16-e71713-s001.docx]

**Table S1 Comparison of Key Mechanisms Between Rat CLBP-D Models and Human Patients**

| Key Mechanism | Rat LDD-Induced CLBP-D Model | Human CLBP-D Patients | References |
| --- | --- | --- | --- |
| NR3C1 methylation rate (PBMCs) | 28% ^[14,22]^ | 45% ^[14,22,27]^ | [14,22,27] |
| Spinal NMDAR NR2B phosphorylation | 1.8-fold increase ^[7]^ | 2.5-fold increase ^[7,13]^ | [7,13,14] |
| Fecal *Prevotella* abundance | ~16% (vs 25% healthy; ↓35%) ^[34]^ | ~13% (vs 21% healthy; ↓40%) ^[34]^ | [34,35] |
| CSF IL-6 level | 2.1-fold increase ^[14,18]^ | 3.2-fold increase ^[18,24]^ | [14,18,24] |

**Table S2 Long-Term Efficacy of Key Therapeutic Interventions for CLBP-D**

| Treatment Type | Specific Intervention | Follow-Up Duration | Long-Term Efficacy (Primary Outcome) | Potential Reason for Efficacy Decline | References |
| --- | --- | --- | --- | --- | --- |
| Pharmacotherapy | Duloxetine (60 mg/day) | 12 months | Pain relief rate 38% (vs. 40% at 8 weeks) | May be related to 5-HT transporter upregulation (25%) | [41,43] |
| Neuromodulation | Closed-loop SCS | 12 months | Response rate 55% (vs. 68% at 3 months) | Is associated with ACC–insula connectivity re-enhancement (32%) | [59,61] |
| Acupuncture | Electroacupuncture (BL23+LR3) | 6 months | Recurrence rate 22% | Is speculated to involve fecal *Prevotella* reduction | [67,68] |

**Table S3 Comparison of Therapeutic Recommendations with International Guidelines**

| Treatment Type | Specific Intervention | Recommendation Level (This Study) | Zhou T et al. ^[77]^ | Cheng C et al. ^[78]^ | Applicable Subtype |
| --- | --- | --- | --- | --- | --- |
| Pharmacotherapy | Duloxetine (SNRI) | First-line | First-line | First-line | All subtypes, especially 5-HT/NE deficiency type |
| Pharmacotherapy | Duloxetine + Minocycline | Emerging (high inflammation) | — | — | Neuroinflammatory subtype |
| Pharmacotherapy | Adalimumab (TNF-α inhibitor) | Second-line (high inflammation) | Second-line (with inflammatory markers) | Second-line | Neuroinflammatory type (serum TNF-α >8 pg/mL) |
| Pharmacotherapy | Esketamine nasal spray | Second-line (refractory) | Not recommended | Second-line (refractory MDD) | Refractory type with NMDAR hyperactivity |
| Neuromodulation | rTMS (DLPFC+ACC) | Second-line | Second-line | Second-line | Cortical-limbic hypersensitivity type |
| Neuromodulation | Closed-loop SCS | Second-line | Second-line | Second-line | Spinal pain-dominant type |
| Acupuncture | Electroacupuncture (BL23+LR3) | Complementary (first-line for mild cases) | Third-line | Complementary | Gut–brain axis type, HPA axis type |

*Note: The treatment recommendations in this table are summarized based on global clinical guidelines for low back pain and regional consensus for major depressive disorder^[77-78]^*
